# Subclinical *Burkholderia pseudomallei* Infection Associated with Travel to the British Virgin Islands

**DOI:** 10.3201/eid2712.211816

**Published:** 2021-12

**Authors:** Courtney M. Dewart, Francisco A. Almeida, Christine Koval, Scott Nowicki, Jay E. Gee, Mindy Glass Elrod, Christopher A. Gulvik, Johanna S. Salzer, Sietske de Fijter, Lindy Liu

**Affiliations:** Centers for Disease Control and Prevention, Atlanta, Georgia, USA (C.M. Dewart, J.E. Gee, M.G. Elrod, C.A. Gulvik, J.S. Salzer, L. Liu);; Ohio Department of Health, Columbus, Ohio, USA (C.M. Dewart, S. Nowicki, S. de Fijter);; Cleveland Clinic, Cleveland, Ohio, USA (F.A. Almeida, C. Koval)

**Keywords:** Burkholderia pseudomallei, Burkholderia, melioidosis, molecular epidemiology, bacteria, British Virgin Islands, travel medicine

## Abstract

Phylogenetic analysis of a clinical isolate associated with subclinical *Burkholderia pseudomallei* infection revealed probable exposure in the British Virgin Islands, where reported infections are limited. Clinicians should consider this geographic distribution when evaluating possible infection among persons with compatible travel history.

*Burkholderia pseudomallei* is a gram-negative aerobic bacillus and the etiologic agent of melioidosis (*1*). The clinical signs and symptoms of melioidosis are varied, and subclinical infection can occur with or without latent clinical manifestation (*1*–*3*). Infection with *B. pseudomallei* typically is associated with environmental exposure through inhalation or direct contact with contaminated soil or water (*1*,*3*). The incubation period can vary from a few days in acute infection to months or years in latent infection, making identification of the exposure source challenging (*1*). Most melioidosis cases are reported in northern Australia and Southeast Asia; however, the known and predicted geographic distribution of *B. pseudomallei* continues to be characterized (*1*,*3*,*4*). We report identification of subclinical *B. pseudomallei* infection by endobronchial ultrasound–transbronchial needle aspiration. We show that phylogenetic analysis of the clinical isolate combined with patient interview were integral to determining a probable location of exposure because the patient traveled to multiple *B. pseudomallei*–endemic regions. This project was reviewed by the Centers for Disease Control and Prevention (CDC) and determined to be nonresearch.

In 2018, a female Ohio resident >65 years of age underwent tooth and torus mandibularis removal after several months of recurrent maxillary molar tooth pain and infections. An oral ulceration was noted, and a biopsy proved it was a squamous cell carcinoma. During her evaluation to undergo maxillectomy and hard palate resection, combined positron emission tomography–computed tomography imaging demonstrated a fluorodeoxyglucose-avid precarinal station 4R lymph node and fluorodeoxyglucose avidity in the right hard palate, consistent with her known malignancy. The patient reported some discomfort at the right upper palate and a sore throat but otherwise had a preserved appetite and weight and denied any chest pain, dyspnea, hemoptysis, fever, chills, or night sweats. She underwent an endobronchial ultrasound–transbronchial needle aspiration, at which time the 4R node was sampled a dozen times. Because a rapid onsite cytology examination failed to demonstrate any malignant cells, additional samples were obtained for routine gram, fungal, and acid-fast bacilli stains and cultures. Scant colonies of *B. pseudomallei* grew on culture media several days after the bronchoscopy, and preliminary identification was made by using VITEK 2 (bioMérieux, https://www.biomerieux.com). 

Results from automated systems in clinical laboratories can misidentify *B. pseudomallei* as a variety of other bacteria and are not confirmatory for this bacterium. Even 16S rRNA gene sequencing can be inadequate depending on the segment queried (*1*). The Ohio Department of Health Laboratory confirmed *B. pseudomallei* by using CDC’s Laboratory Response Network algorithm (https://emergency.cdc.gov/lrn/index.asp). 

Because the patient could not tolerate optimal eradication therapy (*5*), she received intensive therapy with intravenous meropenem for 14 days, then completed a 3-month course of oral doxycycline. Computed tomography images shortly after completing the treatment course showed no evidence of active infection.

During interviews with public health officials, the patient reported traveling to the British Virgin Islands (BVI) twice a year for ≈3 weeks at a time and had visited 2–3 months before the identification of lymphadenitis. She also reported trips of <1 month duration to China and Singapore, where *B. pseudomallei* is endemic, within the previous 10 years (*1*,*3*). No known exposures to *B. pseudomallei* were reported. However, she recalled landscaping activities in BVI that resulted in noticeable dust in her residence, but she did not know on which BVI visit this exposure to aerosolized soil occurred.

CDC performed whole-genome sequencing of the patient’s *B. pseudomallei* isolate, OH2018, for comparison to reference genomes that have well-established geographic origins. The isolate’s genome sequence is available at the National Center for Biotechnology Information (https://www.ncbi.nlm.nih.gov) under Bioproject accession no. PRJNA575632. Multilocus sequence typing classified the isolate as sequence type 92, which previously has been observed in several isolates originating from the Western Hemisphere (*6*,*7*). Phylogenetic single-nucleotide polymorphism analysis demonstrated OH2018 groups with reference genomes from the Caribbean, especially the US Virgin Islands and BVI (Figure).

**Figure:**
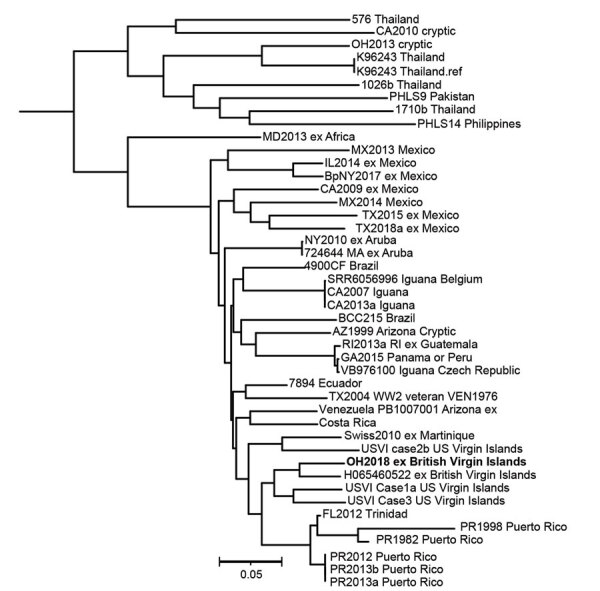
Dendrogram of *Burkholderia pseudomallei* isolated in a patient who traveled to the British Virgin Islands, 2018. Bold text indicates patient isolate; reference genomes predominantly are from the Western Hemisphere. The tree was generated by using MEGA 7.0 software (http://www.megasoftware.net). Single-nucleotide polymorphism analysis was performed by using Parsnp in the Harvest 1.3 package (https://github.com/marbl/harvest). Scale bar indicates nucleotide substitutions per site.

Whole-genome sequencing of the isolate was essential to determining potential exposure risk because the patient traveled to multiple regions where *B. pseudomallei* is endemic. The patient likely was exposed to *B. pseudomallei* in BVI 2–3 months before infection was identified, as ascertained through molecular epidemiology and supported by her report of travel and exposure to aerosolized soil in this location. The case provides additional evidence that *B. pseudomallei* is endemic to the Caribbean and, more specifically, BVI, where reported infections are limited. Only 1 other infection associated with BVI has been reported in the literature (*8*), and no environmental isolates have been reported. To support prompt identification and treatment for melioidosis, clinicians and public health officials should be aware of this geographic distribution when considering possible infection among persons with compatible travel history.

## References

[1] Wiersinga WJ, Virk HS, Torres AG, Currie BJ, Peacock SJ, Dance DAB, et al. Melioidosis. Nat Rev Dis Primers. 2018;4:17107. 10.1038/nrdp.2017.107 29388572 PMC6456913

[2] Chakravorty A, Heath CH. Melioidosis: An updated review. Aust J Gen Pract. 2019;48:327–32. 10.31128/AJGP-04-18-4558 31129946

[3] Currie BJ. Melioidosis: evolving concepts in epidemiology, pathogenesis, and treatment. Semin Respir Crit Care Med. 2015;36:111–25. 10.1055/s-0034-1398389 25643275

[4] Limmathurotsakul D, Golding N, Dance DA, Messina JP, Pigott DM, Moyes CL, et al. Predicted global distribution of *Burkholderia pseudomallei* and burden of melioidosis. Nat Microbiol. 2016;1:15008. 10.1038/nmicrobiol.2015.8 27571754

[5] Lipsitz R, Garges S, Aurigemma R, Baccam P, Blaney DD, Cheng AC, et al. Workshop on treatment of and postexposure prophylaxis for *Burkholderia pseudomallei* and *B. mallei* Infection, 2010. Emerg Infect Dis. 2012;18:e2. 10.3201/eid1812.120638 23171644 PMC3557896

[6] Gee JE, Gulvik CA, Elrod MG, Batra D, Rowe LA, Sheth M, et al. Phylogeography of Burkholderia pseudomallei Isolates, Western Hemisphere. Emerg Infect Dis. 2017;23:1133–8. 10.3201/eid2307.161978 28628442 PMC5512505

[7] Gee JE, Gulvik CA, Castelo-Branco DSCM, Sidrim JJC, Rocha MFG, Cordeiro RA, et al. Genomic diversity of *Burkholderia pseudomallei* in Ceara, Brazil. MSphere. 2021;6:e01259–20. 10.1128/mSphere.01259-20 33536328 PMC7860993

[8] Corral DM, Coates AL, Yau YC, Tellier R, Glass M, Jones SM, et al. *Burkholderia pseudomallei* infection in a cystic fibrosis patient from the Caribbean: a case report. Can Respir J. 2008;15:237–9. 10.1155/2008/290412 18716683 PMC2679542

